# Accuracy of CT Scan for Detecting Hollow Viscus Injury in Penetrating Abdominal Trauma

**DOI:** 10.1007/s00268-023-06954-1

**Published:** 2023-03-01

**Authors:** Anika Wolmarans, Pascaline N. Fru, Maeyane S. Moeng

**Affiliations:** 1 Department of Surgery, School of Clinical Medicine, Faculty of Health Sciences, University of the Witwatersrand, 7 York Road, Parktown, Johannesburg, 2193 South Africa; 2 Trauma Surgery Department, Charlotte Maxeke Johannesburg Academic Hospital, 17 Jubilee Road, Parktown, Johannesburg, 2193 South Africa

## Abstract

**Background:**

In penetrating abdominal trauma, computed tomography (CT) is routinely performed to evaluate stable patients for selective non-operative management (SNOM). Triple-contrast CT (oral, rectal, and IV) has traditionally been used. However, due to its disadvantages, most trauma centres, including our unit at the Charlotte Maxeke Johannesburg Academic Hospital (CMJAH), now perform single-contrast intravenous-only CT scans. We performed a retrospective review to determine the accuracy of single-contrast CT scans for detecting hollow viscus injuries (HVI) in penetrating abdominal trauma.

**Methods:**

A retrospective review of all patients who presented to CMJAH with penetrating abdominal injuries was performed between 01 August 2017 and 31 August 2019 and were evaluated for SNOM with CT (IV contrast only). Patient records were reviewed to determine pertinent demographics, mechanism, and site of injury, as well as metabolic parameters. CT findings were compared to findings at laparotomy.

**Results:**

A total of 437 patients met the inclusion criteria. The majority were male (92.7%), with a mean age of 31.5 yrs (SD 8.7). Injuries were predominantly due to stab wounds (72,5%, *n* = 317). CT scan was negative in 342 patients, of which 314 completed SNOM successfully. A total of 93 patients proceeded to laparotomy. CT had a sensitivity of 95.1%, specificity of 44.2%, positive predictive value of 57.4%, and negative predictive value of 92%.

**Conclusion:**

Single-contrast CT in penetrating abdominal trauma is a valuable investigative tool in identifying patients for SNOM. Features of HVI on single-contrast CT are not very specific and should be interpreted along with other clinical factors including wound trajectory and serial abdominal examinations. Other associated injuries such as diaphragmatic and solid organ injuries should be considered in the final management plan.

## Introduction

Penetrating trauma is the leading cause of abdominal trauma in South Africa, with blunt trauma accounting for 10% of injuries [1]. Stab wounds are the most common cause, attributing to up to 73% of injuries [1–3].

Penetrating abdominal trauma management has moved from mandatory explorative laparotomy to a more conservative approach. Stable patients are now routinely evaluated with computed tomography (CT) to identify patients for selective non-operative management (SNOM) [4].

Several local and international studies have established the safety and efficacy of SNOM for penetrating abdominal trauma [1–8]. It has also been found to be cost-effective in a resource-constrained South African hospital [9]. Patients with haemodynamic instability, peritonism, evisceration, and impalement proceed directly to surgery as current guidelines recommend [8, 10, 11].

Triple-contrast CT (intravenous (IV), oral, and rectal contrast) has been proven to be accurate in identifying hollow viscus injuries (HVI), defined as injuries sustained anywhere along the gastrointestinal tract [12, 13]. However, it has been shown that there is no diagnostic benefit in the addition of enteric contrast for detecting HVI compared to an IV contrast-only CT. In the trauma setting, single-contrast CT is often performed, as triple-contrast CT results in delayed diagnosis and surgical intervention due to the time required for enteric opacification [4, 14, 15].

In recent studies, single-contrast CT has a sensitivity of 75–88% and specificity of 48–100%. However, these studies were limited by small numbers and study design [4, 13, 15].

Stable patients with penetrating abdominal trauma are routinely evaluated with single-contrast CT by the Trauma Unit at Charlotte Maxeke Johannesburg Academic Hospital (CMJAH), Johannesburg, South Africa. Based on CT findings, we have performed several non-therapeutic and negative laparotomies in patients with suspected HVI. Therefore, we aim to determine if there is a significant correlation between single-contrast CT scan and laparotomy findings in the SNOM of penetrating abdominal trauma.

### Study objectives

The primary aim of this study is to determine the accuracy of CT for detecting HVI in penetrating abdominal trauma. The secondary aims are:

1.To determine the most sensitive and specific feature of HVI on CT.

2.To identify the most common injury resulting in failure of SNOM.

## Material and methods

Permission to use the trauma database was obtained from the hospital CEO and the head of the trauma unit of the CMJAH. Ethics approval was obtained from the University of Witwatersrand’s Human Research Ethics Committee (HREC), with clearance certificate number M201029.

A retrospective review was performed for all patients who presented to CMJAH Trauma Unit with penetrating abdominal injuries between 01 August 2017 and 31 August 2019 and were evaluated for SNOM with CT (IV contrast only). Patients who were excluded were those less than 18 years of age, those who required emergency laparotomies, those with low levels of consciousness (GCS score ≤ 8/15), and those who refused further hospital treatment.

Patients who required emergency laparotomy and were excluded were those with haemodynamic instability, peritonitis, evisceration (visceral or omental), pneumoperitoneum on X-ray, and impalement. Haemodynamic instability was defined as a systolic blood pressure < 90 mmHg. Abdominal injuries were defined as injuries sustained within the boundaries of the 5th intercostal space superiorly, the pubic symphysis, inguinal ligament, and iliac crest inferiorly, and the mid-scapular line posteriorly.

Medibank resuscitation forms were used to identify all patients who met the inclusion criteria. CT scan reports were reviewed for features of HVI by either a radiology consultant or a trauma consultant. Patient records were reviewed for relevant clinical features, metabolic parameters, and to determine whether the patient completed SNOM, failed SNOM, or the findings at laparotomy. Theatre records were queried for all patients who underwent laparotomy to determine the presence or absence of an HVI. The presence of one or more of the following features was considered a positive CT scan: pneumoperitoneum, extraluminal air locules, a focal area of bowel wall discontinuity, segmental bowel wall thickening, mesenteric fatty stranding, or a localized fluid collection.

SNOM was successful if the patient completed 24 hours of abdominal observation post-injury and did not require a laparotomy. Worsening abdominal pain, peritonitis, or clinical features of sepsis resulting in laparotomy were considered failure of SNOM.

### Data analysis

Findings at laparotomy were used as the reference standard. Patients with CT features of HVI, and confirmed HVI at laparotomy, were defined as true-positive cases. Statistical analysis was performed using Stata statistical software (version 16.1). Fisher’s exact test and Mann–Whitney test were used to determine the statistical significance of categorical and continuous variables, respectively. A *p*-value < 0.05 was considered statistically significant. Logistic regression was performed to determine accuracy.

## Results

A total of 437 patients met our inclusion criteria; of these, 93 underwent laparotomy.

### Demographics

The majority of patients were male (*n*=405, 92.7%). Most patients were between the ages of 25 and 36 yrs, with a mean age of 31.5 yrs (SD 8.7), and sustained stab wounds (*n*=317, 72.5%).

The injuries sustained were grouped into either isolated abdominal injuries or injuries sustained at multiple sites. The distribution of injuries was almost equal, with 214 (49%) isolated abdominal injuries. The abdominal injury was further described as either anterior, posterior, lateral, or numerous injuries at a combination of these sites. Most patients sustained anterior (164 patients, 37.5%) and posterior (150 patients, 34.2%) injuries. Table 1 summarizes the patient demographics reviewed.

**Table 1:** Patient demographics

Demographics *N* = 437	
*Sex n (%)*	
Male	405 (92.7)
Female	32 (7.3)
*Age (yr)*	
Mean (range)	31.5 (18–68)
*Mechanism of injury n (%)*	
Stab	317 (72.5)
GSW	119 (27.2)
Both (stab and GSW)	1 (0.2)
*Injury n (%)*	
Abdominal	214 (49)
Multiple sites	223 (51)
*Location of abdominal injury*	
Anterior	164 (37.5)
Posterior	150 (34.2)
Lateral	49 (11.2)
Multiple areas	74 (16.9)

*GSW* gunshot wound

A total of 95 (21.7%) patients had features of HVI, and 342 (78.3%) had no features of HVI on CT scan. Of the 342 (78.3%) patients, 314 (91.8%) completed SNOM successfully. Only three patients failed SNOM. A total of 25 (7.3%) patients underwent laparotomy for other indications, including diaphragm injuries, suspected HVI due to wound trajectory, or peritoneal free fluid in the absence of a solid organ injury. Of these 25 patients, 23 had no HVI at laparotomy.

**Fig. 1:**
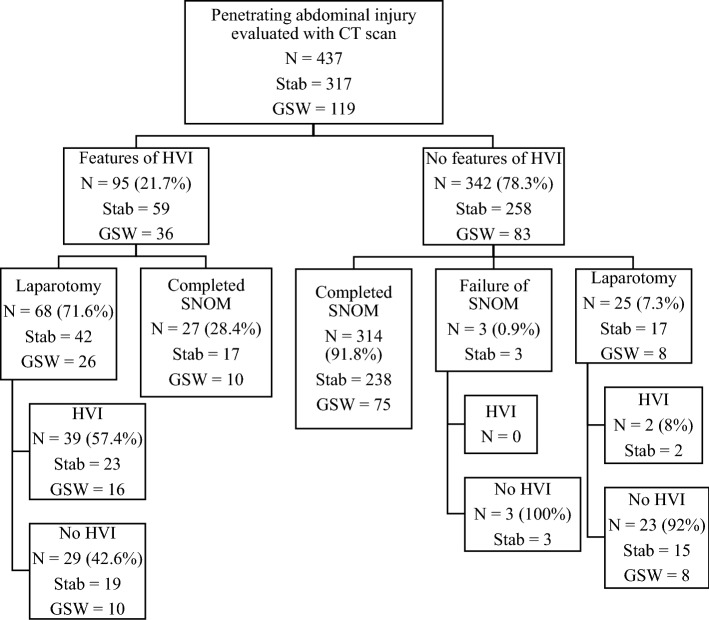
Study population distribution. CT, computed tomography; GSW, gunshot wound; HVI, hollow viscus injury; and SNOM, selective non-operative management

Based on features of HVI on CT scan, 68 (71.6%) patients underwent laparotomy, of which 39 (57.4%) had an HVI. A total of 27 (28.4%) patients with features of HVI on CT scan completed SNOM successfully. Figure 1 is a consort diagram showing the distribution of the patients.

### Bivariate analysis

Bivariate analysis was performed for the 93 patients who underwent laparotomy. Sex and age did not influence the likelihood of sustaining an HVI. Mechanism of injury, whether isolated or multiple injuries, and the location of the abdominal injury did not influence the possibility of an underlying HVI. Clinical features and metabolic parameters evaluated were not statistically significant between the two groups. No specific feature could be identified as sensitive for HVI due to the numerous combinations in which these features occurred. Table 2 indicates the demographic and clinical features evaluated for patients who underwent laparotomy.

**Table 2:** Variables relevant to patients who underwent laparotomy

Variables relevant to patients who underwent laparotomy N = 93					
	No HVI N = 52 (%)	HVI N = 41(%)	Total N = 93(%)	Pearson Chi-Square	*P*-value
*Sex*					1.000
Male	49 (94.2)	39 (95.1)	88 (94.6)		
Female	3 (5.8)	2 (4.9)	5 (5.4)		
*Age (yr)*					0.8911
Range	18–62	20–63	18–63		
Mean	31.8	31.6	31.7		
*Mechanism of injury*				0.1921	0.661
Stab	34 (65.4)	25 (61)	59 (63.4)		
GSW	18 (34.6)	16 (39)	34 (36.6)		
*Injury*				1.1849	0.276
Abdominal	30 (57.7)	19 (46.3)	49 (52.7)		
Multiple sites	22 (42.3)	22 (53.67)	44 (47.3)		
*Location of abdominal injury*				3.1920	0.363
Anterior	16 (30.8)	19 (46.3)	35 (37.63)		
Posterior	14 (26.9)	6 (14.3)	20 (21.5)		
Lateral	9 (17.3)	6 (14.6)	15 (16.1)		
Multiple areas	13 (25)	10 (24.4)	23 (24.7)		
*Pulse (bpm)*					0.6070
Range	64–131	53–117	53–131		
Mean	88.4	85.7	87.2		
*SBP (mmHg)*					0.4442
Range	92–172	63–191	63–191		
Mean	128.2	132.0	129.9		
*DBP (mmHg)*					0.2321
Range	41–113	37–123	37–123		
Mean	73.5	77.1	75.1		
*Lactate*					0.3417
Range	0.6–11.8	0.6–12.3	0.6–12.3		
Mean	3.2	3.1	3.2		
*Base Excess*					0.2178
Range	− 15.3–1.6	− 15.6–4.3	− 15.6–4.3		
Mean	− 4.8	− 3.9	− 4.5		
*Bicarbonate*					0.1614
Range	11.9–27.2	10.3–36	10.3–36		
Mean	20.3	21.6	20.9		

*HVI* hollow viscus injury, *GSW* gunshot wound, and *bpm* beats per minute

### Regression analysis

A total of 52 patients had no HVI at laparotomy. Of these, 23 (44.2%) had no features of HVI (true-negative), and 29 (55.8%) had features of HVI (false-positive) on CT scan. Hollow viscus injuries were found in 41 patients who underwent laparotomy, this confirmed the findings on CT scan for 39 (95.1%) patients (true-positive) and identified injuries not detected by CT scan in 2 (4.9%) patients (false-negative). Table 3 shows the accuracy of single-contrast CT scan.

**Table 3:** Accuracy of CT scan for detecting hollow viscus injury in penetrating abdominal trauma

Measurement	% (95% CI)
Sensitivity	95.12% (83.5–99.4%)
Specificity	44.23% (30.5–58.7%)
Positive predictive value	57.35% (44.8–69.3%)
Negative predictive value	92.00% (74.0–99.0%)

95% CI 95% confidence interval

## Discussion

In keeping with the international and local literature, the majority of patients who present to our trauma unit are males between the ages of 25 and 36 yrs [1–3, 13, 15]. As demonstrated by the results of this study, stab wounds predominate in South Africa. These findings are supported by several local studies. A recent retrospective review of 4697 trauma patients by Bhana and colleagues at Chris Hani Baragwanath Academic Hospital (CHBAH), a level 1 trauma centre in Johannesburg, also found that males between the ages of 29 and 40 yrs constituted the majority (92.1%) of trauma victims and that stabbings are the most common mechanism of injury (71.8%) [16, 17].

The current recommendation by the World Society of Emergency Surgery is that the use of CT scan, complemented by serial clinical examination, should be used to guide surgical decision-making in the SNOM of penetrating abdominal trauma [8]. Mandatory surgical exploration is no longer routine as it is associated with a 61% non-therapeutic laparotomy rate, and the prevalence of hollow viscus and mesenteric injuries is low (17%). Negative laparotomies are also associated with an 8–40% morbidity rate [18, 19]. In South Africa, where thousands of patients present to the emergency department every month, resources are severely constrained. The emergency department at CHBAH receives approximately 11,100 patients per month, of which 65% is related to trauma. Challenges faced include limited access to emergency theatre and CT scans. Therefore, it is imperative to identify patients who potentially require surgery early, as limited access to theatre further delays surgical intervention [16].

Although triple-contrast CT has been proven to be accurate in identifying HVI in penetrating trauma, IV contrast-only CT scans are routinely performed by the CMJAH Trauma Unit [12, 13, 20]. A major disadvantage of enteric contrast is the time delay necessary for enteric opacification, which is the rationale for using single-contrast CT, especially in our resource-constrained environment. The time delay can be up to 68 minutes, a delay further compounded by limited access to theatre [21]. Delayed surgical intervention can result in increased morbidity and mortality [4]. Mingoli [22] and colleagues demonstrated that a delay of > 6 hours in the treatment of a blunt HVI is an independent predictor of postoperative morbidity. Other concerns include the risk of aspiration, especially in patients who will proceed to laparotomy [4, 21]. In addition, recent studies found that triple-contrast CT has a sensitivity equal to that of single-contrast CT in detecting HVI and that the addition of enteric contrast did not provide any diagnostic benefit [4, 14, 15]. As a result, most trauma centres, including the unit at CMJAH, have adopted single-contrast CT as the standard modality [8].

This study found that single-contrast CT has a sensitivity of 95.1% and a specificity of 44.23%. In the largest review to date by Jawad and colleagues, a lower overall sensitivity of 88% and a higher specificity of 72% were reported [4]. The lower specificity demonstrated by our study could be that most of our patients (72%) sustained stab wounds. Jawad et al. reported that gunshot wounds were responsible for 77% of their injuries and found that the sensitivity and specificity for stab wounds were lower, at 80% and 69%, respectively. Likely because it is more difficult to delineate stab wound tracts on CT compared to gunshot wounds [4, 20]. Secondly, Jawad and colleagues did not clearly describe what features determined a positive CT scan. We included intra-abdominal free air and fluid, which are non-specific, indirect features of an HVI, as they could also be due to peritoneal violation. The presence of only these findings on CT can result in high false-positive rates [13]. Another contributing factor to the low specificity of our study is that the majority of patients in this study had negative CT scans and were observed successfully. As there were no operative findings as the reference standard, these patients were excluded from the statistical analysis.

The majority of patients, 314 (71.9%), in this study had negative CT scans and proceeded to complete SNOM successfully. Furthermore, another 27 patients, in which an HVI was clinically unlikely based on the wound trajectory or the absence of abdominal pain, completed SNOM successfully. These results emphasize the importance of clinical evaluation and serial abdominal examinations. Only three patients who had negative CT scans and were selected for SNOM failed observation, none of which had an HVI at laparotomy. As a result, we were unable to identify the most common injury resulting in failure of SNOM. Two of these patients had persistent abdominal pain for more than 24 hours. The findings at laparotomy confirmed the CT findings which were a grade 2 liver injury with haemoperitoneum and a grade 2 liver injury with a grade 2 diaphragm injury, respectively. The third patient developed tachycardia, tachypnoea, and temperature spikes. At laparotomy, a zone 2 retroperitoneal haematoma was found. Subsequently, the patient was diagnosed with pneumonia. No HVI was missed in these patients, and CT accurately detected associated injuries. These results support the findings of Dayananda and colleagues that in select patients, SNOM is a safe strategy in the South African setting [9].

Direct signs of HVI include bowel wall defects and metallic fragments in the lumen or bowel wall. Indirect signs include bowel wall thickening or abnormal enhancement, mesenteric stranding, extraluminal air, and free fluid without solid organ injury [4, 8]. We found that extraluminal air locules, fluid collections, and pneumoperitoneum are often a result of air locules tracking along the wound tract or associated diaphragmatic injuries. Due to the small number of patients who proceeded to laparotomy and the numerous combinations of features of HVI on CT, we were unable to identify the most sensitive and specific feature suggestive of an HVI. However, Saksobhavivat and colleagues found that gastrointestinal wall thickening was the most accurate (accuracy 91%) feature of HVI, and the most sensitive feature is a penetrating wound tract up to the bowel wall (sensitivity 81–98%) [12].

Research into clinical features and investigations that can aid the early diagnosis of HVI is mostly performed in blunt abdominal trauma. In a 2017 prospective study by Matsumoto and colleagues, they evaluated the use of intestinal fatty acid-binding protein (I-FABP) levels in aiding the early diagnosis of HVI in blunt abdominal trauma. They also evaluated other clinical parameters and found that the heart rate, systolic blood pressure, and lactate levels were not significantly different between the groups with and without an HVI. Only abdominal tenderness and an elevated I-FABP level were statistically significant. Several other studies showed similar results, confirming that clinical examination together with a CT scan of the abdomen plays a vital role in the early diagnosis of an HVI [23–26]. In keeping with these studies, none of the clinical features, location of injuries, or metabolic parameters we evaluated were statistically significant. Data from patients who suffered injuries at multiple sites and those who suffered isolated abdominal injuries were analysed together, which potentially influenced our results and is one of this study’s limitations.

Another limitation to our study is that statistical analysis was performed only for the patients who underwent surgical exploration, as a reference standard is required. The majority of patients were treated non-operatively, and the false-positive and true-negative cases may have been underestimated. Secondly, we may have missed patients who failed SNOM and presented after 24 hours or to other hospitals, underestimating the false-negative rate. Lastly, our retrospective study design, conducted at a single institution, may be open to incomplete reporting and potential treatment practice bias.

Other studies within our context have evaluated and proven the safety of clinical evaluation and CT scan in the SNOM in penetrating abdominal trauma [1, 9, 27, 28]. However, our unit is the first to evaluate the accuracy of CT scan for detecting HVI in penetrating abdominal trauma in our environment.

Further research opportunities would be to proceed to a prospective study to identify the most sensitive and specific features of HVI on single-contrast CT and to determine which section of the intestine is the most sensitive.

## Conclusion

Single-contrast CT in the setting of penetrating abdominal trauma is a valuable investigative tool in identifying patients for SNOM. Features of HVI on single-contrast CT are not very specific and should be interpreted along with other clinical factors including wound trajectory and serial abdominal examinations. Other associated injuries such as diaphragmatic and solid organ injuries should be considered in the final management plan.
